# Dataset on the calculations of daily adult word and conversational turn counts, and use of styles of oral interaction in 2–5-year olds with hearing loss in New Zealand

**DOI:** 10.1016/j.dib.2020.105372

**Published:** 2020-03-09

**Authors:** Nuzhat Sultana, Lena L.N. Wong, Suzanne C. Purdy

**Affiliations:** a Division of Speech and Hearing Sciences, Faculty of Education, the University of Hong Kong, Hong Kong; b School of Psychology, the University of Auckland, Auckland, New Zealand

**Keywords:** Natural language input, Quantity of language input, Number of adult words, Number of conversational turns, Styles of oral interaction, Children with hearing loss, LENA calculations

## Abstract

This article describes the data regarding the calculations of language input from the natural language environments of children with hearing loss, taken from four full typical days in a week using a LENA (Language ENvironment Analysis) digital recorder. Calculations were based on 14 children with hearing loss from 24 to 60 months as they interacted with their family. Participants were recruited from the Hearing House, the Speech Clinic at the University of Auckland, and Early Childcare Centers (ECC) in Auckland, New Zealand. All families were interacting with their children orally without using sign language. Data were collected from natural language environments from May 2018 to May 2019. Language environments were examined in terms of daily quantity of language input and styles of oral interaction children were exposed to when interacting with their parent/primary caregiver. To determine quantity of language input, two kinds of observations were taken from the LENA automatic calculation of the number of adult words and number of conversational turns. Segments of the recordings were manually transcribed and coded onto 17 styles of oral interaction, which were further classified into three categories (optimal, moderate, and sub-optimal).

**Specifications Table**

**Table:** 

**Subject**	Social Sciences
**Specific subject area**	Linguistic and Language
**Type of data**	Table
**How data were acquired**	Language ENvironment Analysis (LENA) digital recorder and LENA software
**Data format**	Raw
**Parameters for data collection**	Calculations of number of adult words and conversational turns were determined using automatic LENA software. A count of styles of oral interaction was extracted by manual transcription and coding of LENA recordings for 10 min/day (i.e., 5 min in the morning between 8:30 a.m. and 11:30 a.m., and 5 min in the evening between 4:00 p.m. and 8:00 p.m.) when the LENA graph showed the highest number of conversational turns for the individual child. The LENA software separates each 5-minute segment of recording automatically. Seventeen styles of oral interaction (i.e., comments, ‘*wh*’ & ‘yes/no’ questions, expansion, recast, labeling, directives, etc.) were classified into three major categories (optimal, moderate, and sub-optimal). Language abilities were assessed according to the assessment protocols of the Preschool Language Scale-Fifth Edition
**Description of data collection**	LENA recordings were obtained from four full typical days selected by the parent/caregiver (two weekend days and two weekdays when the child was mostly with the parent/caregiver) from morning to evening. The parent/caregiver was instructed to not include preschool days and days where unusual events such as family gatherings were taking place. All families were informed that the child would wear a comfortable vest with a pocket to carry the LENA recorder for the full typical day. They were advised to turn on the LENA recorder in the morning as early as possible when the child woke up and to turn it off at night when the child went to bed. They were instructed to turn off the LENA recorder and remove the vest during bath or nap time. The families completed recordings according to the instructions when the family was not engaged with special occasions, such as birthday parties, family get together etc. The families were also instructed that they should behave naturally interacted with their children as usual during the recording days. There were no restrictions for the parents on engaging in usual activities such as staying home, shopping, visiting a playground, or having a picnic at the beach. Due to privacy concerns, the families were informed that their child's identity (e.g., name of child and/or date of birth) would not be shown anywhere. Also, they could withdraw their participation at any time during the data collection process if they felt uncomfortable with the recording due to an unusual day or they could stop recording anytime of the day
**Data source location**	Auckland, New Zealand
**Data accessibility**	Data is with this article

**Value of the Data**

• Calculations of quantity of language input (number of adult words and conversational turns) from four typical days during natural interactions including the number of the use of 17 different styles of oral interaction during parent/caregiver to child communication exchanges enhances the evidence base for parent-child oral interactions in natural settings.
• Data on language input develops our understanding of parental language behaviours and can be used to link input to language outcomes. Few such data have been collected in the past [1].
• These data will enable clinicians to better advise parents/caregivers about how to change their quantity and quality of oral interactions with their young children in natural settings [2].
• These data can be used as a reference for the comparison of language input between children with and without hearing loss.

## 1 Data

Table 1 shows the child demographics: gender, age at recording, age at identification, level of hearing loss, type of device use and age when first amplification was received and family information: the reported time which the parent/caregiver usually spent with a child during weekdays and weekend days, number of adults who shared the house at the same time and interacted with the child daily, number of siblings, child's birth order, and parental level of education. According to information reported by parents, all 14 children had both parents (father and mother) but usually the primary caregiver was the child's mother.

**Table 1:** Demographic information reported by parent/primary caregiver in 14 children with hearing loss.

Demographics		P1	P2	P3	P4	P5	P6	P7	P8	P9	P10	P11	P12	P13	P14
Child demographics	Gender	M	M	F	F	M	M	F	M	F	F	F	M	F	F
	Age at recording	26mo	34mo	26mo	57mo	26mo	39mo	26mo	27mo	27mo	53mo	36mo	25mo	48mo	57mo
	Age at identification	1mo	3mo	2mo	3mo	6mo	3mo	5mo	4mo	3mo	3mo	4mo	3mo	4mo	4mo
	Level of hearing loss	Profound	Profound	Profound	Profound	Profound	Severe- Profound	Severe- Profound	Moderate- Severe	Profound	Moderate	Moderate	Moderate- Severe	Moderate- Severe	Moderate- Severe
	Type of device (bilateral)	CI	CI	CI	CI	CI	CI	CI	HA	HA	HA	HA	HA	HA	HA
	Age first received amplification	7mo	6.5mo	6mo	14mo	6mo	14mo	6mo	3mo	6mo	6mo	5mo	6mo	6mo	4mo
Family demographics	Parental time spend with child/weekday	6h	6h	4h	6h	8h	10h	5h	5h	6h	6h	5h	5h	5h	6h
	Parental time spend with child/weekend day	10 h	10 h	8 h	6 h	14 h	12 h	12 h	10 h	12 h	12 h	12 h	12 h	12 h	12 h
	Number of adults in family	2	3	2	2	4	2	3	2	2	2	3	3	4	4
	Number of siblings	1	3	3	2	2	4	3	3	2	2	3	1	2	2
	Child's birth order	1	2	2	2	2	3	2	3	2	2	3	1	1	2
	Mother's education level	8	8	8	6	8	7	7	8	7	7	7	8	6	6
	Father's education level	9	7	7	6	8	8	7	7	7	7	7	7	6	6

*Note:* P = participants; M = male; F = female; mo = months; CI = cochlear implant; HA = hearing aid; BE = both ear; h = hour. Parental Level of education was defined as the New Zealand education classification system: 10 = Doctoral degree, 9 = Master degree, 8 = Bachelors honors, 7 = Bachelors, 6 = A certificate for technical knowledge within a specific field.

Table 2 shows the recorded time for each recording, and the automatic LENA calculations (total number of adult words and total number of conversational turns per day) for individual participant.

**Table 2:** Total number of recorded hours per day and calculations of number of adult words, and conversational turns for each day two weekend days (WE) and two weekdays (WD) in 14 children with hearing loss.

Recorded time and quantity of input	Days	P1	P2	P3	P4	P5	P6	P7	P8	P9	P10	P11	P12	P13	P14
Duration of recordings per day	WE1	14h:13m	14h:10m	13h:33m	13h:46m	13h:59m	14h:24m	13h:29m	13h:49m	13h:49	13h:59m	14h:13m	14h:00m	13h:27m	14h:24m
	WE2	09h:39m	13h:39m	14h:06m	13h:33m	13h:59m	13h:44m	14h:19m	10h:14m	13h:12m	14h:00m	14h:11m	14h:00m	13h:39m	13h:45m
	WD1	12h:38m	14h:35m	13h:40m	13h:43m	14h:20m	13h:01m	14h:21m	12h:38m	13h:14m	13h:45m	13h:00m	14h:24m	13h:29m	13h:44m
	WD2	14h:05m	14h:19m	13h:07m	13h:52m	13h:55m	13h:12m	13h:45m	13h:05m	14h:00m	14h:00m	13h:59m	14h:00m	13h:38m	14h:00m
Total number of adult words	WE1	32456.65	22494.02	12154.35	10325	19,380.9	7750.08	9117.43	31543.45	13860.88	14346.9	11967.59	13020	7852.11	12873.6
	WE2	21087.18	18304.65	12503.88	6804.81	24456.85	12417.68	20547.28	22361.88	13543.2	18958.8	8101.52	11617.2	4856.67	11566.5
	WD1	13378.7	16336.25	14637	16764.51	1526	7442.93	8050.35	13378.7	14506.38	12185.25	11130.6	11387.52	11625.33	15639.52
	WD2	24868.35	19602.38	11427.24	13794.56	17593.45	10240.56	14338.5	23102.55	13104	11281.2	12752.8	10474.8	6732.14	12264
Total number of conversational turns	WE1	682.4	1190	552.84	346.92	1023.58	578.88	266.97	663.2	845.58	520.18	784.76	562.8	403.5	501.12
	WE2	677.43	794.43	482.22	308.94	1283.67	799.28	111.67	718.38	673.2	1024.8	953.12	571.2	245.7	684.75
	WD1	439.64	892.5	598.6	477.34	1247	624.8	215.25	439.64	794	536.25	390	432	671.47	535.6
	WD2	785.85	919.13	393.5	316.16	960.25	617.76	272.25	730.05	865.2	420	604.08	445.2	384.46	394.8

*Note*: P = participants; h = hours; m = minutes.

Table 3 shows manual calculations of the number of adult words, and conversational turns for 10 min segments (two × 5 min) extracted from each recording/each day for two weekend days and two weekdays in 14 children with hearing loss. Forty minutes of recording (two 5 min/day) was extracted for each participant. The LENA pro-software version (V3.4.0-143) automatically identified 5 min intervals with the highest number of adult words and conversational turns during the time periods from 8:30 a.m. to 11:30 a.m. and 4:00 p.m. to 8:00 p.m..

**Table 3:** Calculations of number of adult words, and conversational turns for 10 min segments extracted from the recordings for each day two weekend days (WE) and two weekdays (WD) in 14 children with hearing loss.

Quantity of input	Days	P1	P2	P3	P4	P5	P6	P7	P8	P9	P10	P11	P12	P13	P14
Total number of adult words for 10 min	WE1	1333	1216	775	817	1132	444	304	1333	722	939	442	433	550	1360
	WE2	1143	879	861	560	1192	685	974	1143	1134	1011	271	725	592	775
	WD1	899	1167	1315	784	848	461	484	899	474	710	356	329	434	551
	WD2	873	1255	966	541	740	1318	380	873	265	1252	312	305	364	988
Total number of conversational turns for 10 min	WE1	50	48	32	12	50	38	21	49	45	65	45	21	22	38
	WE2	55	51	31	19	60	32	19	55	45	60	37	44	37	21
	WD1	61	45	48	22	41	35	20	71	32	54	20	32	38	18
	WD2	41	47	38	14	54	49	22	41	39	16	23	22	22	30

*Note*: P = participants.

Table 4 shows the total number of 17 styles of oral interaction that were extracted from the 10 min LENA recorded segments of conversational turns for manual transcription and coding. Six styles of oral interaction under the ‘optimal’ category were extracted: comment, open-ended questions, positive marker, recast, expansion, and reason, four ‘moderate’ (close-ended question, labeling, repetition, action) and seven ‘sub-optimal’ (joint speech, directive, one-word response e.g., yes/no/ok, linguistic mapping, imitation, negative markers) styles of interaction, respectively. The scores indicate the total number of times each style of oral interaction was used over the two 5 min periods per day. Results are shown separately for the four typical days. During these times children were engaged in meals, playing with toys, and dressing/clothing.

**Table 4:** Calculations of number of 17 styles of oral interaction for each day two weekend days (WE) and two weekdays (WD) in 14 children with hearing loss.

Styles of oral interaction		Days	P1	P2	P3	P4	P5	P6	P7	P8	P9	P10	P11	P12	P13	P14
Optimal Styles of Interaction	Comment	WE1	18	14	24	04	15	05	07	17	06	10	11	12	05	06
		WE2	08	25	04	00	14	10	03	14	16	12	11	10	06	06
		WD1	18	22	23	10	14	11	05	20	15	08	10	12	13	08
		WD2	26	19	12	09	31	09	12	17	07	08	12	10	08	12
	Open-ended question	WE1	09	04	03	02	01	03	01	08	01	01	06	07	02	04
		WE2	08	02	03	01	05	10	02	11	05	10	07	10	07	03
		WD1	18	01	09	06	04	10	00	07	10	03	06	04	05	05
		WD2	07	08	09	05	09	08	02	02	02	04	03	03	05	04
	Positive marker	WE1	02	00	02	00	07	00	00	01	04	01	01	01	01	04
		WE2	00	01	01	01	09	08	03	06	03	04	02	02	01	01
		WD1	02	02	01	01	04	00	00	01	07	04	02	00	03	01
		WD2	00	05	04	01	07	02	00	01	01	00	03	01	05	05
	Recast	WE1	00	00	04	00	00	00	00	00	00	00	00	00	00	00
		WE2	02	01	00	00	04	00	00	02	00	00	00	00	00	00
		WD1	04	01	02	00	00	00	00	00	00	00	00	00	00	00
		WD2	00	02	02	00	00	00	00	00	00	00	00	00	00	00
	Expansion	WE1	01	01	02	00	03	01	00	03	00	01	01	00	00	00
		WE2	03	08	04	00	05	00	01	01	01	01	01	00	00	01
		WD1	06	05	03	01	03	00	01	02	00	01	01	00	01	02
		WD2	03	04	03	07	09	00	01	01	00	00	01	01	00	00
	Reason	WE1	06	04	00	00	02	00	00	03	00	02	00	00	00	03
		WE2	11	00	02	00	03	00	00	01	03	01	00	06	01	01
		WD1	05	03	00	01	01	01	00	01	05	01	00	00	05	04
		WD2	06	05	01	04	03	01	01	04	04	02	00	00	01	05
Moderate Styles of Interaction	Close-ended question	WE1	05	11	10	05	06	07	07	10	07	07	09	12	07	06
		WE2	00	08	13	02	25	00	02	21	07	17	10	09	09	15
		WD1	10	08	05	10	11	05	05	15	00	06	11	08	12	05
		WD2	10	22	09	07	21	11	03	07	10	11	10	09	04	05
	Labeling	WE1	00	00	07	09	00	01	00	01	02	02	02	04	00	04
		WE2	07	00	10	00	03	00	03	04	00	02	02	05	01	01
		WD1	03	04	00	01	01	01	04	01	01	04	01	08	03	05
		WD2	01	04	10	00	01	02	01	00	05	13	04	04	00	03
	Repetition	WE1	00	00	02	00	04	01	00	00	03	00	02	01	02	02
		WE2	05	04	00	01	12	05	03	02	00	02	03	00	01	01
		WD1	01	07	01	04	10	02	02	01	01	00	02	00	01	00
		WD2	00	03	00	03	02	05	05	02	03	02	03	01	02	02
	Action	WE1	01	01	01	01	02	00	00	03	00	00	03	02	00	02
		WE2	01	04	06	01	01	01	03	00	02	01	01	02	01	01
		WD1	01	00	00	00	00	01	00	01	03	02	03	00	01	05
		WD2	00	03	01	01	01	01	00	00	01	02	04	00	00	00
Sub-optimal Styles of Interaction	Joint speech	WE1	00	00	00	00	00	00	00	02	00	00	00	00	00	00
		WE2	00	00	03	00	00	03	00	00	00	00	00	00	00	00
		WD1	00	00	00	00	03	00	01	00	00	00	00	00	00	00
		WD2	00	02	00	02	00	00	00	00	00	00	00	00	00	00
	Directive	WE1	16	11	14	07	12	09	12	21	07	08	16	08	06	13
		WE2	04	25	07	03	22	18	03	19	06	19	21	07	07	11
		WD1	14	25	06	17	15	14	06	17	21	20	18	09	22	12
		WD2	12	20	15	12	33	19	23	22	06	09	16	13	06	18
	One word response	WE1	00	03	02	00	04	03	13	04	03	04	03	02	03	03
		WE2	07	00	03	01	08	03	06	05	05	02	01	02	02	00
		WD1	01	11	04	02	02	02	02	02	01	08	04	00	12	04
		WD2	00	06	02	01	06	04	02	00	01	04	01	03	08	07
	Lingusitic mapping	WE1	00	00	00	00	00	00	00	00	00	00	00	00	00	00
		WE2	00	00	00	00	00	00	00	00	00	00	00	00	00	00
		WD1	03	00	00	00	00	00	00	00	00	00	00	00	00	00
		WD2	01	00	01	00	00	00	00	00	00	00	00	00	00	00
	Imitation	WE1	00	00	03	02	08	02	00	00	00	01	02	02	02	04
		WE2	06	02	02	01	02	03	01	06	00	01	03	00	02	01
		WD1	05	03	03	04	02	07	00	01	05	01	01	00	01	02
		WD2	00	05	01	02	03	00	00	05	01	02	00	00	01	01
	Negative marker	WE1	00	01	01	04	03	01	00	02	05	05	04	03	02	02
		WE2	12	03	00	02	01	01	06	03	04	04	04	07	01	05
		WD1	01	03	01	00	01	04	03	00	03	02	05	03	08	05
		WD2	00	06	01	03	05	03	03	00	02	00	04	05	01	02
	Others	WE1	00	01	02	00	02	00	04	03	01	03	01	00	00	02
		WE2	02	03	01	00	21	01	03	02	03	05	00	00	00	04
		WD1	00	05	02	00	00	00	00	01	02	04	00	01	04	04
		WD2	00	00	02	00	07	00	00	01	02	02	02	00	00	01

Table 5 provides the descriptions and examples for each style of oral interaction coded for the data set. Table 6 shows each child's receptive and expressive language scores used to investigate the link between language input and outcomes.

**Table 5:** Styles of oral interaction and classification of the three main categories with the detail description and examples.

Main categories	Styles Oral of Interaction	Description	Examples
Optimal Styles of Oral Interaction	Comment	The parent attempts to make a statement or phrase as a signal that the message has been received or to keep their conversation going.	The parent says, “you are working hard” or “you saw this book before.”
	Open-ended question	Using a simple “Wh” question and a phrase or sentence as a simple justification for the child to give an answer using more than two words.	The parent asks, “What is that?” or “why are you interested in listening to this story?”
	Positive marker	The parent shows verbal excitement about the child's action using words.	The parent says “alright,” “great,” “good job,” “well done,” “nice,” “pretty work,” etc.
	Recast	The parent rephrases the child's vocalization as a question.	The child says, “Anna went …” and the mother says, “Where did Anna go?”
	Expansion	The parent repeats the child's verbalization and completes it accurately using a more grammatical and complete language model with the addition of one or more words, without adding new information.	The child says, “Doggie goes …” and the parent says, “The dog is going.” Or the child says, “Baby cry …” and the parent says, “The baby is crying,” etc.
	Reason	The parent attempts to give a specific explanation regarding their verbal interaction.	The parent says, “You should try to wash your hands because you are big now.”
Moderate Styles of Oral Interaction	Closed-ended question	The parent makes a statement to which the child can only answer with one word.	The parent says, “Do you want to go to the park?” or “do you need water?”
	Labeling	The parent indicates the name of the animal, building, road, fruit, object, etc.	The child asks, “What's that?” The mother says, “The moon,” “a lady,” “a sticker,” “a pond,” “a bird,” etc.
	Repetition	The parent attempts to repeat sounds, words, and sentences to draw the child's attention to a statement or verbal command, without adding new words or information.	The parent says “sh, sh, sh,” or “water, water,” or “it's tasty, it's tasty.”
	Action	The parent uses statements with action verbs.	The parent says, “He is walking,” “stars are shining,” etc.
Sub-Optimal Styles of Oral Interaction	Joint speech	The parent and child speak together while reading, rhyming, and singing.	The parent and child speak at the same time, “knees and toes, knees and toes,” etc.
	Directive	The parent gives a direct command to the child to do something.	The parent says, “Come here,” “listen carefully,” “read the word,” “sit down,” hold it,” etc.
	One word response	The parent uses only one word to answer the child.	The parent says “yes,” “no,” “yeah,” “okay,” “right,” etc.
	Linguistic mapping	The parent attempts to create word-based information based on the child's unrecognizable vocalization.	The child vocalizes “wa, wa” and the parent says “water.” Or the child says, “hoda hoda” and the parent says “hiding.”
	Imitation	The parent imitates the child's vocalization without adding new words.	The child says, “a choc-bar” and the parent repeats “a choc-bar.”
	Negative marker	The parent responds negatively to the child's verbal attempts.	The parent says, “No, that's not right,” “very bad,” etc.
	Other	The parent gives an answer to the child in an improper form of language.	The parent says “hmmm,” “hahaha,” “umm,” “uh,” “oh,” “oop.”

**Table 6:** Receptive and expressive language scores in 14 children with hearing loss.

Language outcome	P1	P2	P3	P4	P5	P6	P7	P8	P9	P10	P11	P12	P13	P14
PLS-5 receptive language standard scores	82	96	80	74	74	70	74	70	74	102	72	56	70	70
PLS-5 expressive language standard scores	80	96	80	74	73	62	74	62	74	99	72	54	70	70

*Note:* P = participants; Preschool Language Scale-Fifth Edition (PLS-5).

## 2 Experimental design, materials, and methods

The Language ENvironment Analysis (LENA) system was used for recordings and automatic calculations of natural language input: a) number of adult words, and b) number of conversational turns. Recordings for four typical days (two weekdays, two weekend days) were collected. Quantity of language input (number of adult words, and number of conversational turns), ranged from 9 h 39 min to 14 h and 24 min each day.

To identify the frequency of 17 styles of oral interaction between parent/caregiver and child each day the four days LENA recordings were used. In total 40 min of recording segments were extracted for each participant for four typical days (two x 5 min per day, one morning and one evening). Age standard scores of receptive and expressive language abilities were obtained using PLS-5 [3].
